# Effects of elevated CO_2_ levels on lung immune response to organic dust and lipopolysaccharide

**DOI:** 10.1186/s12931-021-01700-4

**Published:** 2021-04-09

**Authors:** David Schneberger, Upkardeep Pandher, Brooke Thompson, Shelley Kirychuk

**Affiliations:** College of Medicine, University of Saskatchewan, Saskatoon, SK Canada

**Keywords:** Carbon dioxide, Lung, Organic dust, LPS, Inflammation, Innate immunity

## Abstract

Workplaces with elevated organic dust levels such as animal feed barns also commonly have elevated levels of gasses, such as CO_2_. Workers exposed to such complex environments often experience respiratory effects that may be due to a combination of respirable factors. We examined the effects of CO_2_ on lung innate immune responses in mice co-exposed to the inflammatory agents lipopolysaccharide (LPS) and organic dust. We evaluated CO_2_ levels at the building recommended limit (1000 ppm) as well as the exposure limit (5000 ppm). Mice were nasally instilled with dust extracts or LPS and immediately put into chambers with a constant flow of room air (avg. 430 ppm CO_2_), 1000 ppm, or 5000 ppm CO_2_ enriched air. Results reveal that organic dust exposures tended to show decreased inflammatory responses with 1000 ppm CO_2_ and increased responses at 5000 ppm CO_2_. Conversely, LPS with addition of CO_2_ as low as 1000 ppm tended to inhibit several inflammatory markers. In most cases saline treated animals showed few changes with CO_2_ exposure, though some changes in mRNA levels were present. This shows that CO_2_ as low as 1000 ppm CO_2_ was capable of altering innate immune responses to both LPS and organic dust extracts, but each response was altered in a different fashion.

## Background

Organic dust exposures in animal feed operations has long been shown to be detrimental to the health of workers in these facilities [1, 2]. Experimental approaches to determining the factors present in these dusts that may be responsible for respiratory inflammation have yielded considerable data, particularly on the role of microbial products such as endotoxins like lipopolysaccharides (LPS) and proteoglycans, and others as potential causes of these lung problems [3, 4]. Such dust exposures however are often in the context of facilities with a variety of elevated gases. Ammonia and hydrogen sulfide have been implicated in causing lung problems or being immuno-modulatory [5, 6]. However, carbon dioxide (CO_2_) has often been overlooked despite significant data showing routine elevations of CO_2_ in animal feed operations [7].

Carbon dioxide levels in animal confinement operations often exceed standards including the American Society of Heating, Refrigeration and Air-conditioning Engineers (ASHRAE) recommended limit (1000 ppm) as well as the Occupational Safety and Health Administration (OSHA) 8 h time weighted average (TWA) limit of 5000 ppm. CO_2_ at levels as low as 1000 ppm have been shown to induce cognitive changes in humans [8, 9]. Indeed, elevated CO_2_ is a broader problem, and common in many facilities such as schools [9], daycares [10, 11], prisons [12], cars [13], airplanes [14], and many other locations [15].

Previously we have shown that innate immune responses to organic dust extracts were changed when co-exposed to CO_2_ at 5000 ppm [16]. These changes were at the protein and mRNA levels and in most cases were indicative of enhanced inflammation. Subsequent to this study others showed that neutrophils were altered by similar levels of CO_2_ [17]. At present however these are the only studies we are aware of to address this issue in lung immunology at these low levels. There have been several studies related to behavior [18] and at higher levels of CO_2_ where there is induction of hypercapnic acidosis in animals and cell cultures [19–21]. Results for these latter studies have been somewhat mixed, particularly in situations of LPS exposure versus more complex challenges such as tobacco extracts [21, 22]. These studies also do not address CO_2_ at levels applicable to most work environments and crowded spaces [23, 24].

The aims of the current study were to extend the previous co-exposure model to assess lower levels of CO_2_ exposure while at the same time testing both a defined single inflammatory stimuli (LPS) and a complex one (organic dust extracts). We hypothesize that the lung alters innate immune responses when presented with a combined exposure of an inflammatory agent and CO_2_ as low as 1000 ppm.

## Methods

### Animals

Six to seven week old female C57BL/6 mice were purchased from Charles River Laboratories (Montreal, Quebec, Canada). C57BL/6 mice were selected for purposes of comparability to other papers on organic dust exposure and female mice to be comparable to our previous study on CO2 exposure [16]. Mice were housed in standard cages in a controlled room with temperature of 23 °C ± 2 °C and a 12-h light/dark cycle. Mice were fed standard laboratory chow and water ad libitum. Mice were acclimatized in their first week in facility for 2 h per day for 5 days to whole body plethysmography chambers (part 601-1425-001; DSI, Minneapolis, MN) ventilated by a Buxco Finepointe whole body plethysmography 4-site system (DSI, Minneapolis Minnesota).

Mice were randomly assigned to 9 experimental groups (5 mice/group): each of control (saline) with room air (avg 430 ppm CO_2_), control (saline) with 1000 ppm CO_2_, or control (saline) with 5000 ppm CO_2_; LPS with room air (avg 430 ppm CO_2_), LPS with 1000 ppm CO_2_, or LPS with 5000 ppm CO_2_; or Organic Dust (HDE) with room air (avg 430 ppm CO_2_), Organic Dust (HDE) with1000 ppm CO_2_, or Organic Dust (HDE) with 5000 ppm CO_2_ exposure. All intranasal treatments (saline, LPS, and HDE) were given between 8:00 and 8:30 AM to minimize any potential circadian rhythm differences between treatments and then mice were immediately placed in Buxco Finepoint whole body plesthysmography 4-site system (DSI, Minneapolis, Minnesota) with 1000 ppm or 5000 ppm CO_2_ concentrations, or room air for 6 h. At end of the experiment mice were euthanized.

### Organic dust extracts, LPS, and control treatments

Intranasal treatment with organic dust extracts (HDE), LPS, or control (saline) were done immediately prior to the 6-h gas exposure in plethysmography chambers (see CO2 Delivery and Whole Body Plethysmography).

Organic dust extracts (HDE) were produced from settled dust samples collected from swine confinement facilities. These dust extracts have been characterized previously for muramic acid, endotoxin, and protein [25], and the bacterial composition has been previously described [26]. HDE extracts were prepared as described previously [27] by mixing 1 g dust with 10 ml HBSS (without calcium, Sigma, St. Louis, MS) and incubated for 1 hr at room temperature before centrifugation twice, and subsequent filter sterilization through a Nalgene 0.2 μM SFCA membrane syringe filter (ThermoFisher, Rochester, NY), resulting in a solution of approximately 0.105 g/ml dust. To prevent any variation due to dust used or extraction procedure, all extract samples in this study were created from a single extraction of the dust sample. Dust extracts were given to mice at a final concentration of 12.5% vol/vol or 0.005 g/ml dust delivered in 40 μl dropwise to the nares, and mice allowed to inhale under light (1%) isoflurane anesthesia.

LPS from *Escherichia coli* 0111:B4 (lot 095M4163V, Sigma, St. Louis) was diluted in HyClone HBSS (1X) (HyClone Laboratories; Logan, UT) to 0.1 µg, and delivered in 40 μl to the nares, and mice allowed to inhale under light (1%) isoflurane anesthesia. Control groups were given 40 μl HBSS in the same manner under light anesthesia.

### CO_2_ delivery and whole body plethysmography

All mice upon intranasal treatment were placed immediately into whole body plethysmography (WBP) chambers (DSI, Minneapolis, MN) for the 6-h exposure under positive airflow of 5 psi. The bias flow air consisted of either room air (avg 430 ppm CO_2_), or air from tanks consisting of room air enriched for CO_2_ to 1000, or 5000 ppm CO_2_ (Praxair; Mississauga, Canada). Biasflow was constant through the exposure period to prevent any accumulation of CO_2_ due to respiration from animals. Mice were provided with food and water in exposure chambers for the duration of exposures. CO_2_ readings of the treatment room provided an average CO_2_ level of 430 ppm as measured using a Q-Trak Indoor Air Quality Monitor 7575 (TSI; Shoreview, MN).

Readings from the WBP chambers were captured every 2 s for the entire 6 h duration of exposure. Results were averaged for 20 min intervals using the Finepointe system software (DSI, Minneapolis, MN). Measures of PenH, peak inspiratory volume, peak expiratory volume, volume per minute, and breath frequency were taken.

### Bronchoalveolar lavage

Lungs were lavaged as detailed earlier [27]. Briefly, lungs were washed three times with 0.5 ml HBSS each time. Bronchioalveolar lavage (BAL) fluid was centrifuged at 1000×*g* for 10 min, and supernatant transferred to new tubes and stored at − 80 °C. Cell pellets were resuspended in HBSS and stained with trypan blue (Life Technologies, Grand Island, NY) and counted using a hemocytometer. Cells were resuspended to 100 μl in HBSS and adhered to glass slides (Fisher Scientific; Pittsburgh, PA) via cytocentrifugation for 10 min at 10,000×*g* (Cytopin, Shandon Elliott, Great Britain). Cells were dried overnight and fixed and stained using Diff Quik kit (Siemens Healthcare Disagnostics, Newark, DE) and mounted using MM24 mounting media (Leica Biosystems; Buffalo Grove, IL). A differential count of 100 cells was made based on morphological assessment of cells and expressed as absolute cell numbers.

### Lung collection

After BAL collection lungs were excised. The right lung was tied off at the primary bronchus, removed, flash frozen in liquid nitrogen, and stored at −80 °C. The left lung was slowly inflated with 200 μl of 4% PFA (Sigma; St. Louis, MO) and stored in 4% PFA overnight, followed by 100% ethanol. The fixed lung was embedded the next day in paraffin in a Intelsint RVG/1 tissue processor (Intelsint; Turin, Italy) followed by mounting in paraffin blocks using a Tissue Tek II tissue embedder (Sakura Finetek; Nagano, Japan).

### Lung histology

Lung sections from paraffin embedded lungs were sectioned in 5 μM slices on a Microm 350S microtome (Microm, Germany). Tissue sections were then stained with Hemotoxylin and Eosin using the Histology Core Facility’s Protocol (https://healthsciences.usask.ca/facility-services/histology-core-facility.php) and mounted using Surgipath MM24 (Leica Biosystems, Richmond, IL). Sections were imaged using an Aperio CS2 virutual microscopy system (Leica Biosystems, Concord ON Canada) and examined for any notable changes to lung structure.

### ELISA

Quantification of MCP-1, MIP-2, IL-6, KC, IL-1β, TNF-α, IL-4, IL-5, IL-13, IL-33, and IL-10 in BAL fluid was determined by Luminex xMAP multiplex ELISA (Procartaplex, ThermoFisher) according to manufacturer’s specifications. Samples were read using a Bioplex 200 system (Bio-Rad, Hercules, California) and Bioplex Manager Software (Bio-Rad).

### RNA purification and RT-PCR analysis

RNA was purified from lung tissue samples using a Qiagen RNeasy Plus Mini kit (Qiagen, Chatsworth CA) according to the manufacturer’s instructions for tissue extraction. Briefly, 20–25 mg of lung tissue was put into 350 μl of RLT lysis buffer and 2.0 mm zircon beads (BioSpec, Bartlesville, OK). Samples were placed in a Biospec Mini-BeadBeater-24 (BioSpec) and run twice for 2 min, with a pause between runs of 5 min where samples were held on ice to prevent heat buildup. Supernatant was removed to DNA eliminator column, and the remainder of purification  was done according to the kit protocol. RNA was quantified by Take3 plate (Biotek, Winooski, VT) in a Synergy HT plate reader (BioTek). cDNA synthesis was done using the iScript Reverse Transcription Supermix for RT-qPCR (Bio-Rad, Hercules, CA) with 300 ng of template mRNA. Samples were incubated at 25 °C for 5 min, 46 °C for 20 min, and finally at 95 °C for 1 min in a Bio-Rad CFX96 Touch Real-Time PCR Detection System (BioRad). RT-PCR was done using probes for TLR2 (Mm00442346_m1), TLR4 (Mm445273_m1), Hsp72 (Mm01159846_S1), A20/TNFAIP3 (Mm00437121_m1) (Life Technologies, Grand Island, NY). Ribosomal RNA (Life Technologies) was used as an endogenous control in all reactions. PCR was conducted using a Bio-Rad CFX96 Touch Real-Time PCR Detection System (Bio-Rad). Reactions were all carried out in duplicate and performed 2 min at 50 °C, 10 min at 95 °C, then 40 cycles of 15 s at 95 °C and 1 at 60 °C min each using ddPCR Supermix for Probes kit (Bio-Rad). Relative comparison of selected targets to the ribosomal endogenous control cycle threshold (CT) value was analyzed with the ΔΔCt method.

### Statistical analysis

Data was analyzed using GraphPad Prism 6 (GraphPad Software, San Diego, CA). Error bars represent mean ± SEM. For values outside the assay limit of detection (LOD) either the LOD/2 or a minimum value below the lowest attained value was designated. Statistical significance was determined using one-way ANOVA with follow-up Tukey test for multiple comparisons. If the assumption of equal variance using the Brown’s Forsythe test was not met, the data was log transformed followed by one-way ANOVA and multiple comparison tests. For all tests, a p-value ≤ 0.05 was considered significant for differences between groups.

## Results

### Plethysmography data

**Fig. 1:**
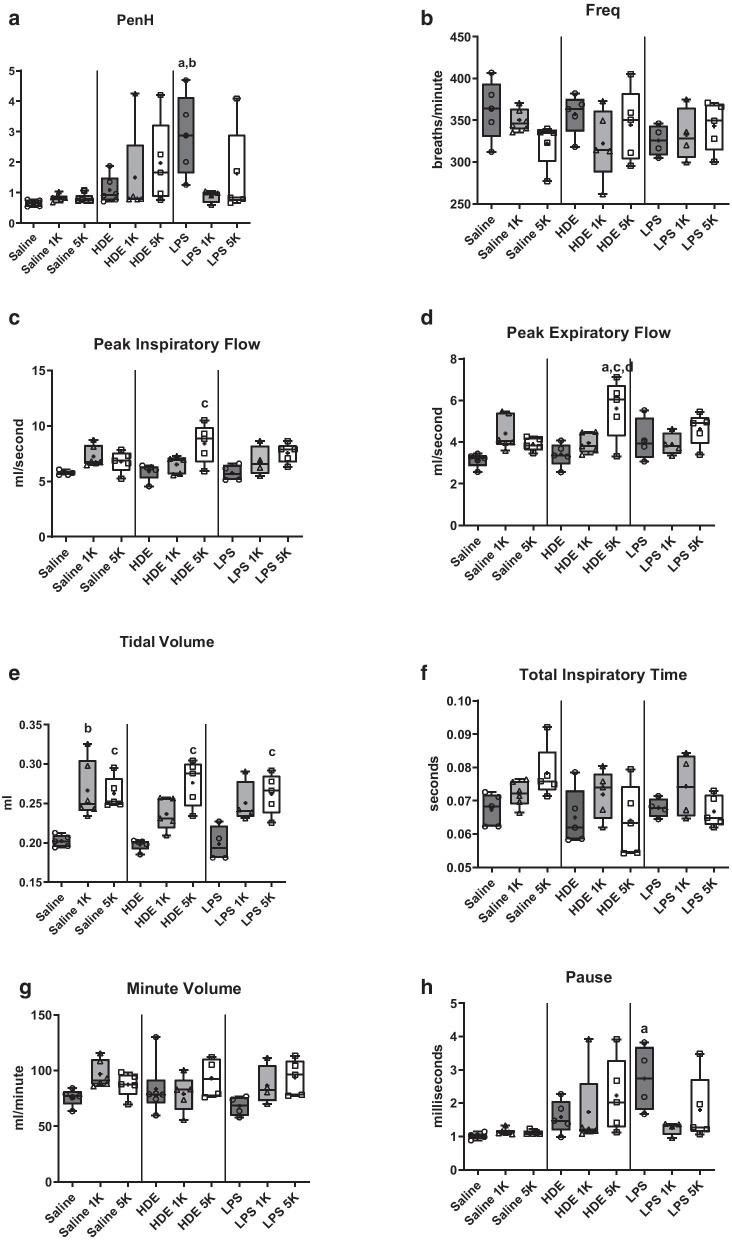
Plethysmography data: Mice were monitored during exposure period and PenH, peak inspiratory volume, peak expiratory volume, volume per minute, and breath frequency. Each result represents the average value over the 6 h exposure. Statistical significance denoted by “a” (treatment vs respective saline control), “b” (1000 ppm CO_2_ vs room air treatment), “c” (5000 ppm CO_2_ vs room air treatment) and “d” (5000 ppm CO_2_ vs 1000 ppm CO_2_ treatment). N = 5 mice/group. Minimum, maximum, mean (**+ **symbol) and median (line) are denoted for each treatment

Monitoring of breathing parameters of mice in exposure chambers showed changes in several parameters over the 6 h of exposure, across all treatments (Fig. 1).

Animals given saline showed elevations in peak inspiratory and expiratory flow (Fig. 1c, d), tidal volume, total volume (Fig. 1e), and minute volume (Fig. 1g) at 1000 ppm CO_2_, and tidal volume and total inspiratory time (Figs. 1e and f) at 5000 ppm. Animals treated with HDE showed significant increased peak inspiratory flow, p < 0.05 (Fig. 1c) and tidal volume, p < 0.01 (Fig. 1e) over room air at 5000 ppm CO_2_, whereas at 1000 ppm only tidal volume was elevated (p < 0.05). LPS treatment to animals showed increases in tidal volume over room air co-exposure (p < 0.05) at 1000 and 5000 ppm CO_2_ co-exposure. CO_2_ meanwhile resulted in a decrease in LPS-induced increase in PenH, (p < 0.01) (Fig. 1a).

Comparing within and between treatments there was increased tidal volume with all co-treatments with CO_2_ compared to treatments with no CO_2_.

None of the treatments appeared to have any significant effect on breathing frequency (Fig. 1b), total inspiratory time (Fig. 1f), or minute volume (Fig. 1g).

### Bronchoalveolar lavage cells

**Fig. 2:**
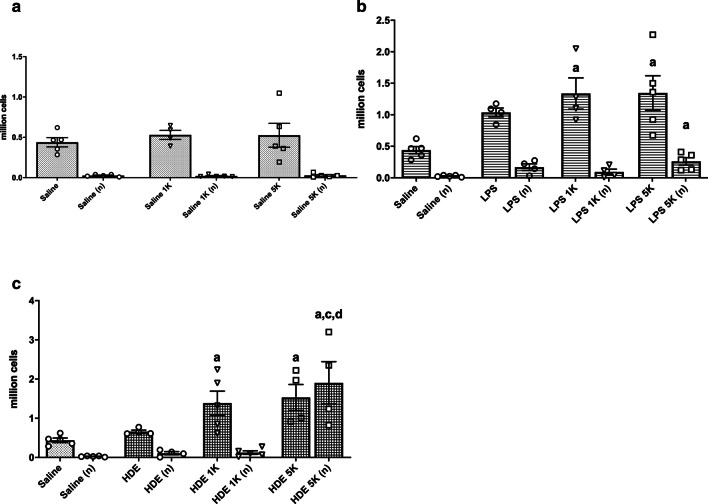
Effects of CO2 on BAL fluid cells. Mice were treated intranasally with saline, organic dust (HDE), or lipopolysaccharide (LPS) and then exposed to either room air (~ 500 ppm), 1000 ppm (1 K), or 5000 ppm (5 K) CO_2_. Values given are based on number of cells in BAL fluid multiplied by the fraction of monocytes/macrophages (black bars) or neutrophils (open bars) in (**a**) saline, (**b**) HDE or (**c**) LPS treated animals. Bar graphs represent averages and error bars the SEM. N = 4–5 mice/group. Statistical significance denoted by “a” (treatment vs respective saline control), “b” (1000 ppm vs room air treatment), “c” (5000 ppm vs room air treatment) and “d” (5000 ppm vs 1000 ppm treatment) and error bars denoting SEM

Cell counts from BAL fluid showed several patterns (Fig. 2). First, mice given saline intranasally showed no significant changes in BAL cell composition or number with increased CO_2_ (Fig. 2a).

LPS treatment showed an increase in macrophages/monocytes compared to saline control. This increase was significant to treatment at a similar CO_2_ level but only at 5000 ppm CO2 co-exposure (p < 0.05). No significant increases in neutrophils were noted between saline and LPS treatments, except between Saline + 5000 ppm CO_2_ and LPS + 5000 ppm CO_2_ (p < 0.01).

HDE exposure (Fig. 2c) on its own or with room air was not able to induce a significant rise in monocytes/macrophages. However, co-exposure of HDE with 1000 ppm CO_2_ was able to induce an non-significant increased numbers of cells over saline given at a similar level of CO2 (p = 0.0732), which was significantly increased with 5000 ppm co-exposure (p < 0.05). Levels of neutrophils were elevated with HDE, but not significantly different as compared to saline. However, co-exposure of HDE with 5000 ppm CO_2_ caused a substantial increase of the neutrophil population as compared to HDE and room air (p < 0.001), which was also significantly different from the HDE and 1000 ppm CO_2_ co-exposure (p < 0.01), and from the saline and 5000 ppm CO_2_ co-exposure (p < 0.0001).

### ELISAs

**Fig. 3:**
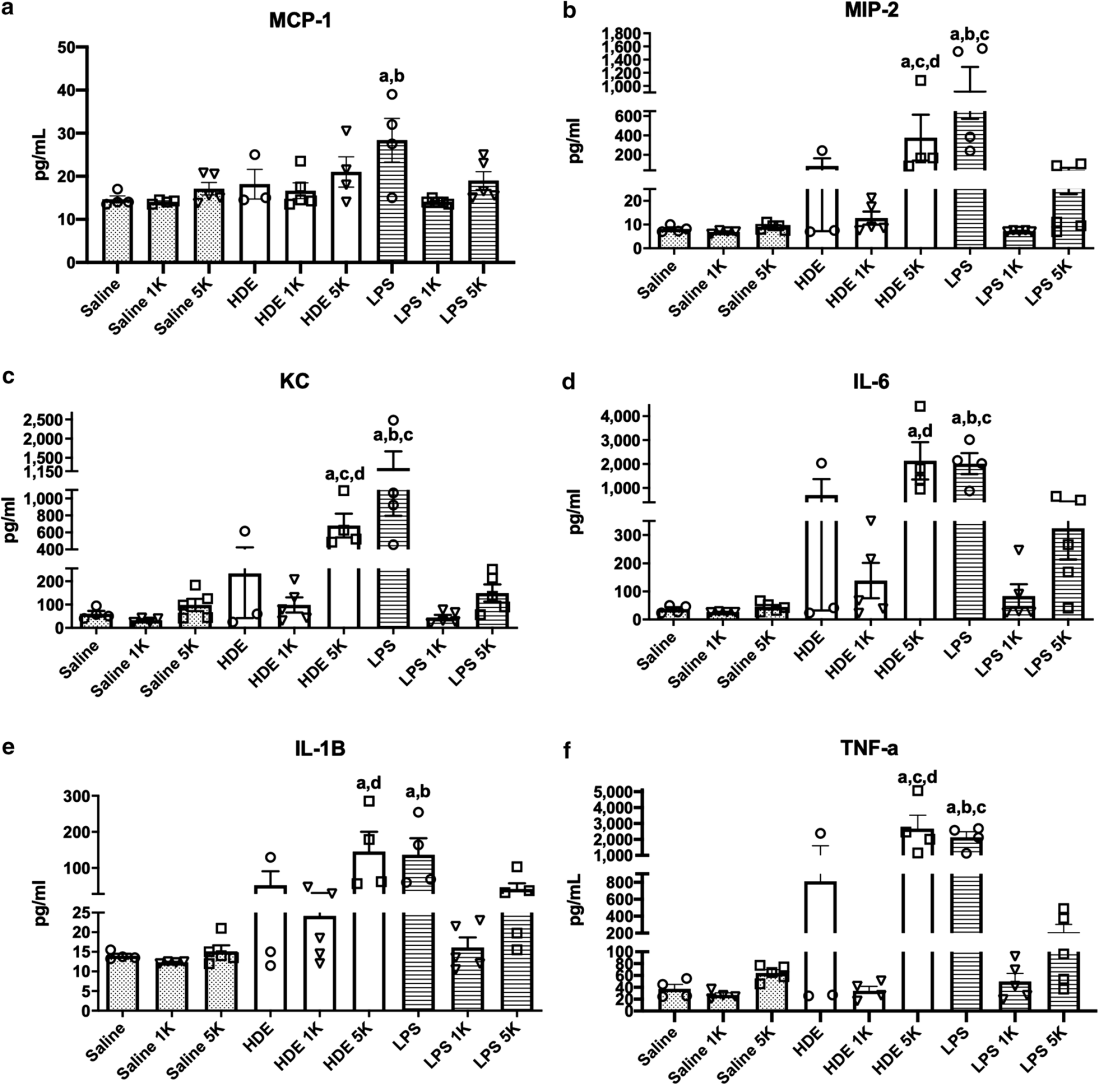

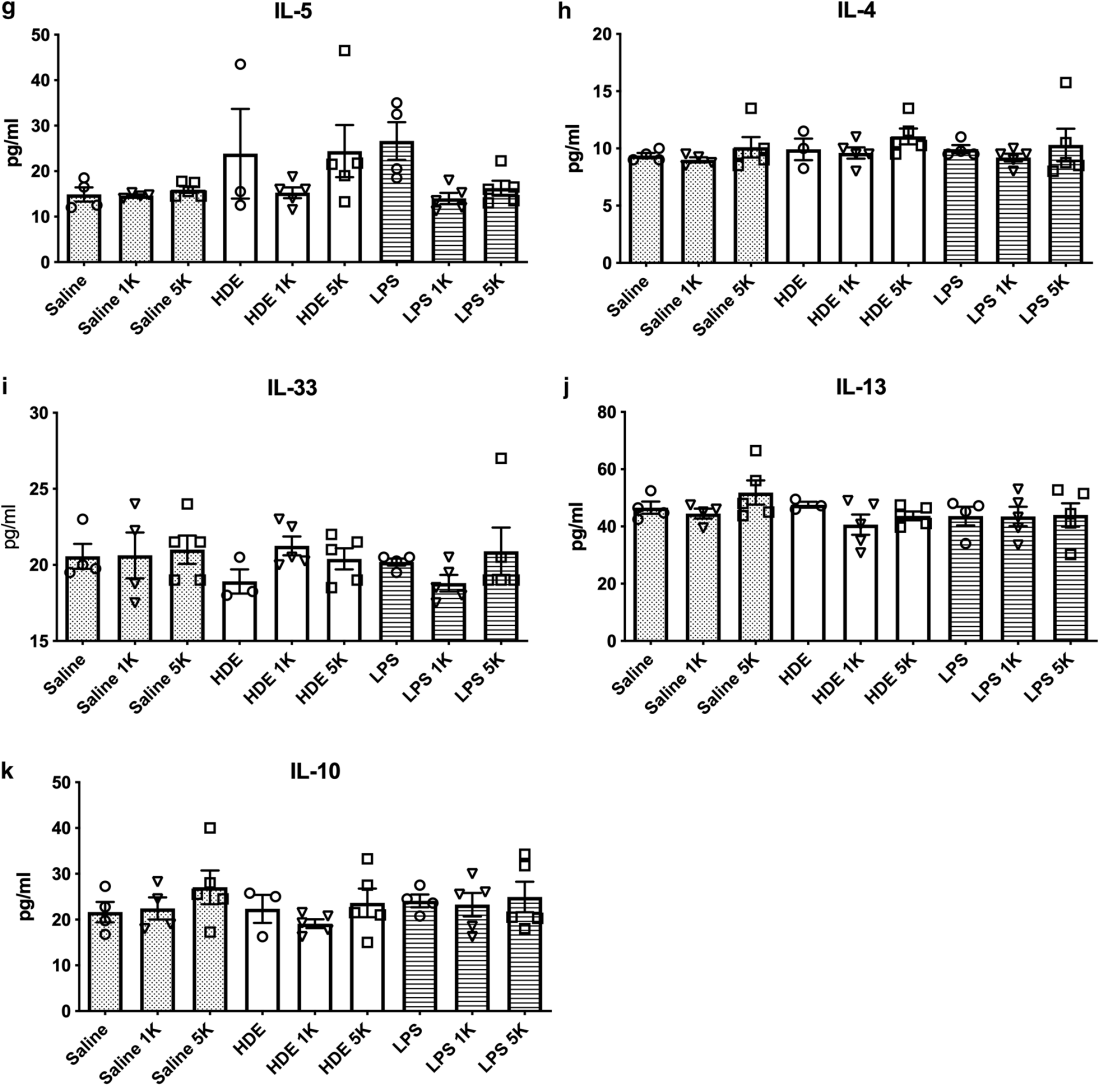
Protein expression in BAL fluid. Values given are in OD reading (**a**) MCP-1, **b** MIP-2, **c** KC, **d** IL-6, **e** IL-1B, **f** TNF-a, **g** IL-5, **h** IL-4, **i** IL-33, **j** IL-13, and **k** IL-10. Mice were treated intranasally with saline, organic dust (OD), or lipopolysaccharide (LPS) intranasally and then exposed to either room air (~ 500 ppm), 1000 ppm (1 K), or 5000 ppm (5 K) CO_2_. BAL fluid was centrifuged, cells removed and tested by ELISA. Results show reductions in KC and MIP-2 with LPS treated animals plus CO_2_, and an increase in all three for OD plus 5000 ppm CO_2_. Bar graphs represent averages and error bars the SEM. N = 3–5 mice/group. by “a” (treatment vs respective saline control), “b” (1000 ppm vs room air treatment), “c” (5000 ppm vs room air treatment) and “d” (5000 ppm vs 1000 ppm treatment)

As shown in Figs. 3–1 and –2, the expression of cytokines appeared to follow three general patterns. First, no difference in cytokines was noted when CO_2_ was co-exposed with saline.

For LPS, increased CO_2_ provoked a decrease in cytokines/chemokines as compared to co-exposure with room air. Co-delivery of 1000 ppm CO_2_ with LPS resulted in significant decreases in IL-6 (p < 0.01), KC (p < 0.001), TNF-α (p < 0.01), and IL-1β (p < 0.01) as compared to co-exposure with room air. Similar trends were noted at 5000 ppm CO_2_ co-exposure with LPS with reductions in IL-6 (p < 0.05), KC (p < 0.001), and TNF-α (p < 0.01) as compared to room air co-exposure to LPS. Cytokine/chemokines were not significantly different between co-exposure of LPS to CO_2_ at 1000 ppm and 5000 ppm as compared to saline co-exposures at these same CO_2_ levels.

The situation was more complex with HDE. Compared to co-exposure with room air, at 1000 ppm CO_2_ co-exposure HDE showed no significant differences between cytokines/chemokines though there was some apparent drop in production. However at 5000 ppm CO_2_ co-exposure there was a significant rise in MCP-1 and MIP-2 (p < 0.05) compared to HDE on room air. There was also a rise in MCP-1 (p < 0.001), MIP-2 (p < 0.01), and IL-1β (P < 0.01) at 5000 ppm codelivery as compared to co-delivery of 1000 ppm CO_2_. This suggested an increase in inflammatory response at higher CO_2_ delivery as opposed to the reductions seen with all CO_2_ co-delivery levels when CO_2_ was co-exposed with LPS. Further, with HDE co-exposure with 1000 ppm CO_2_ there was a tendency to reduced cytokines whereas 5000 ppm CO_2_ co-delivery with HDE showed increases in cytokines, suggesting differential responses with increased CO_2_ concentrations when co-exposed with HDE.

### RT-PCR

We looked for evidence of changes to signaling in lung cells first at the receptor level with two of the receptors most commonly associated with response to HDE, TLR2 and TLR4, the latter of which is critical to responses to LPS.

**Fig. 4:**
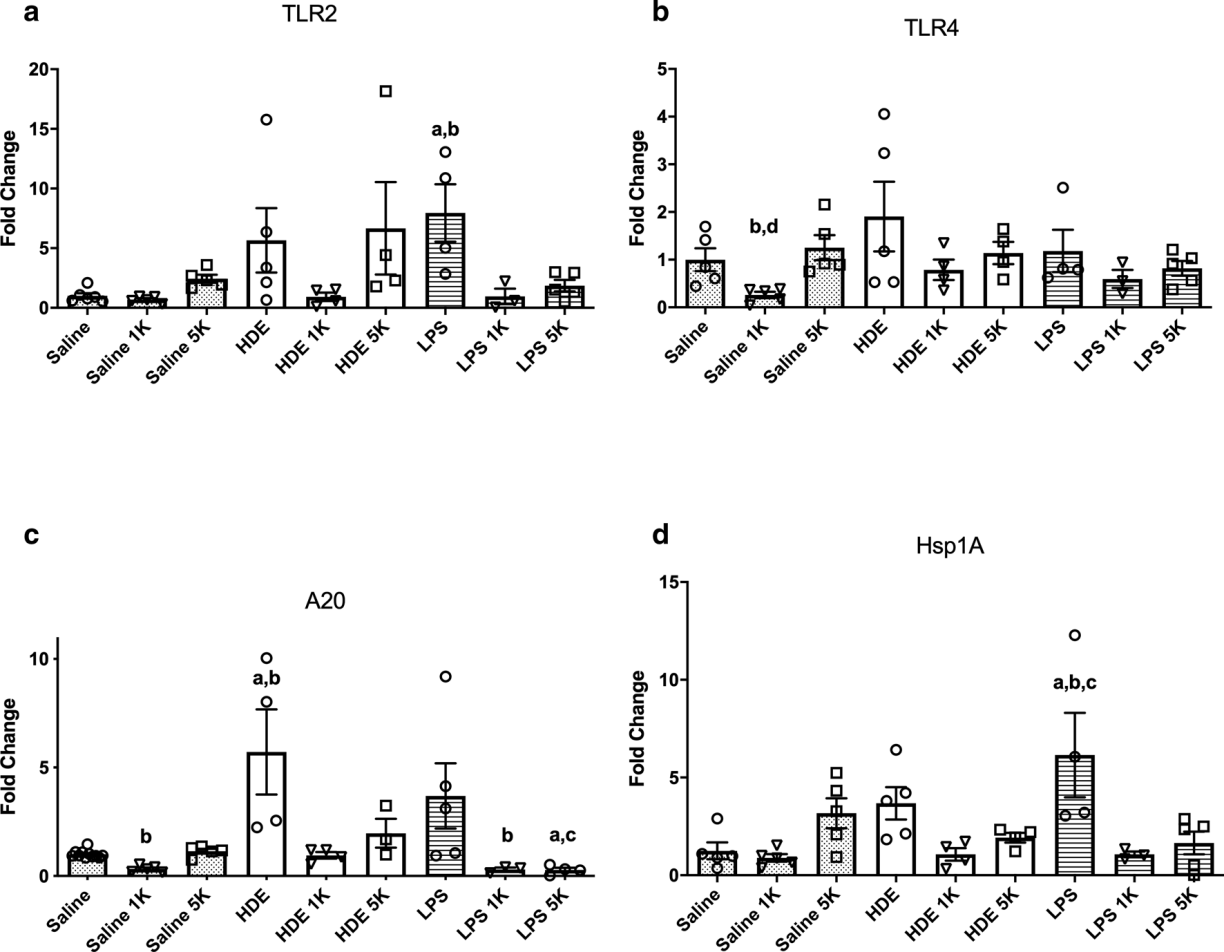
mRNA expression in mouse lung tissue. Values given are fold change in mRNA compared to a control value. mRNA harvested from lungs of mice given saline, organic dust (OD), or lipopolysaccharide (LPS) intranasally and then exposed to either room air (approximately 500 ppm), 1000 ppm (1 K), or 5000 ppm (5 K) CO2. Tissue was tested for the receptor TLR2 (**a)**, TLR4 (**b**), cytoplasmic protein A20 (**c**) and Hsp1A (**d**). Results show reductions in TLR2, and Hsp1A with LPS treated animals plus CO_2_, and general decreases in A20 and Hsp1A for OD plus CO_2_. Hsp1A was elevated with CO_2_ delivery to control, to date the only protein mRNA found to do this. Bar graphs represent averages and error bars the SEM. N = 3–5 mice/group. by “a” (treatment vs respective saline control), “b” (1000 ppm vs room air treatment), “c” (5000 ppm vs room air treatment) and “d” (5000 ppm vs 1000 ppm treatment)

TLR2 (Fig. 4a), which is implicated in HDE induced inflammation [28, 29] showed no significant change with HDE co-exposure with any level of CO_2_. However, LPS with room air induced a significantly large increase in TLR2 mRNA compared to saline and room air (p < 0.05), and this increase was inhibited by addition of 1000 ppm CO_2_ (p < 0.01) and 5000 ppm CO_2_ (NS) co-exposures.

TLR4 (Fig. 4b) showed no significant change in mRNA expression across all LPS and HDE treatments and CO_2_ co-exposures.

With little work having been done on CO_2_ signaling pathways we next looked at a negative regulator of the TLR/NF-kB pathway, TNFAIP3 (A20) (Fig. 4c), which has been implicated by others in the prevention of asthma in rural populations [30]. While there was no increase in mRNA production with LPS exposure, there was an increase in A20 mRNA expression with HDE compared with control (p < 0.01). Interestingly, for HDE, this increase was inhibited by CO_2_ co-delivery at 1000 ppm (p < 0.05), and less inhibitory at 5000 ppm.

mRNA expression of Hsp72 (Hsp1A) was also tested (Fig. 4d).This protein has been shown to have effects on MAPK and NF-kB signaling and thus may alter signaling in these two key pathways. CO_2_ at 5000 ppm co-exposure with saline was able to induce some Hsp72 mRNA increase, but not a significantly compared to saline and room air. When co-exposed with room air, HDE induced a similar non-significant Hsp72 increase while LPS induced a significant increase (p < 0.01). However, Hsp72 mRNA was decreased when HDE or LPS stimuli were co-exposed with either of the two CO_2_ concentration (p < 0.05 for LPS + CO_2_ treated groups).

### Histology

**Fig. 5:**
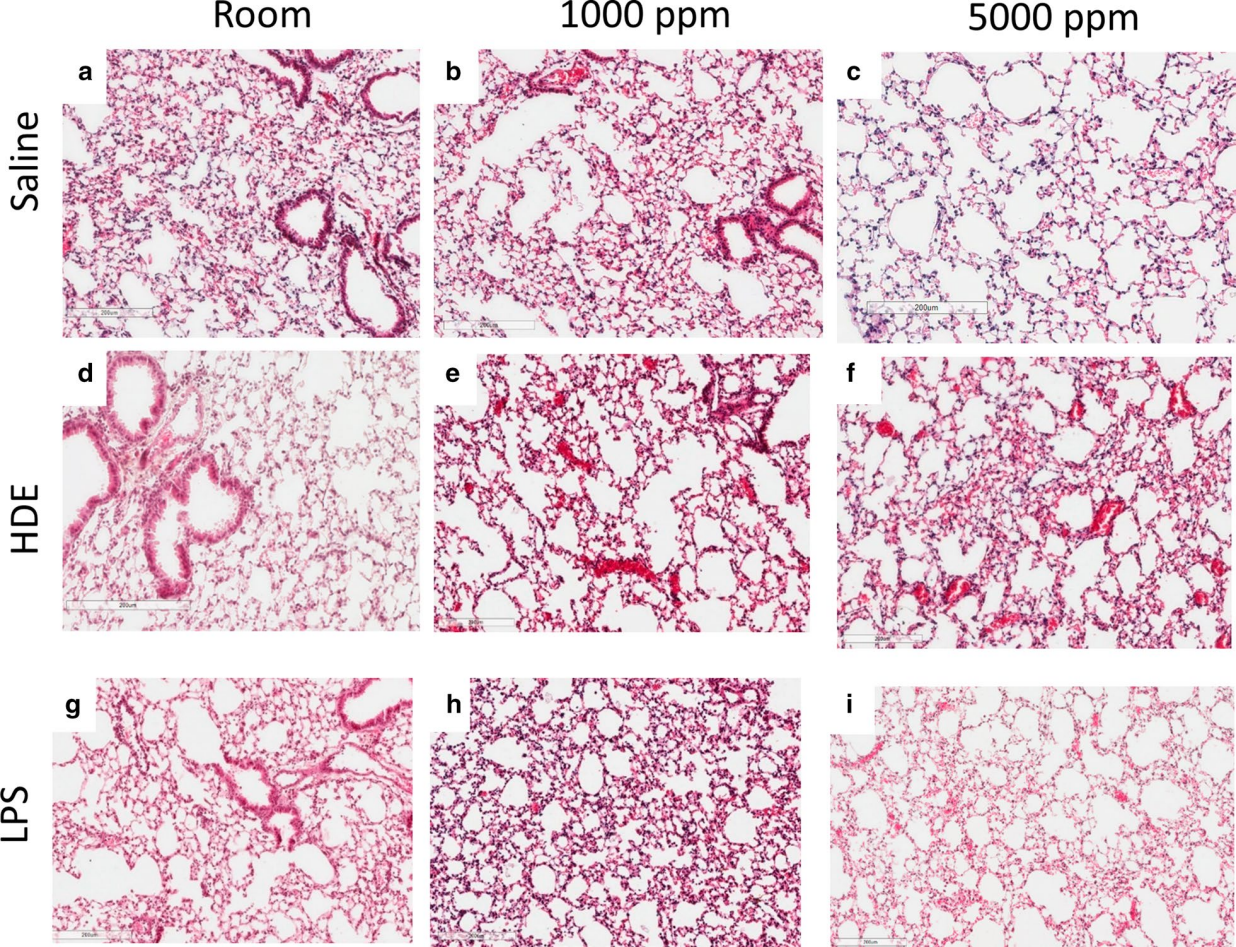
Histological examination of mouse lungs. Left lungs of mice were fixed in 4% PFA and processed for paraffin blocks. 5 μm tissue slices were cut and stained with hematoxylin and eosin and imaged. Images at 100× are included for saline (**a**–**c**), HDE (**d**–**f**), and LPS (**g**–**i**). Changes in inflammation were apparent between saline, HDE, and LPS, treatments, but CO_2_ co-treatment appeared to have little effect

Tissue sections (Fig. 5) overall showed little in the way of overt changes due to inflammation. This may be in part due to the limited time frame of exposure, and longer exposures are likely to produce more significant changes. No consistent overt differences were noted in mice for saline, LPS, or HDE treatments compared to their similarly treated but CO2 co-exposed duplicates.

## Discussion

In high intensity animal production operations, there are buildups of both particulates in the air as well as a variety of gasses, mostly as a result of the animals housed there. Given the problems of respiratory effects in exposed workers [1, 31] numerous studies have tried to recreate these conditions in animal models [2] to determine responsible agents for health effects. Given that CO_2_ elevation is common in many environments, not just in barns, we previously looked at the effects of human workplace safety limit levels (5000 ppm) of CO_2_ on mice treated with barn dust extract to try to devise a more holistic model of barn exposure inflammation. The results from this study suggested that we look at both a more simple innate inflammatory molecule (LPS), as well as CO_2_ concentrations that are more reflective of a number of environments.

The first questions was if breathing parameters showed any changes, as might be expected from an elevation in ambient CO_2_ levels. Martrette et al. have reported potential stress after long repeated exposures to elevated CO_2_ in rats (700 ppm, 6 hr/day over 15 days) [18], whereas Rasid et al. have shown changes in minute volume associated with stressful procedures [32]. In the present study there were no significant changes to minute volume for any of the treated animals over the 6 h course of our studies, suggesting the treatments were reasonably tolerated. Similarly, breath frequency was unaffected across all treatments and CO_2_ levels. Tidal volume appeared to be the most sensitive of the tested parameters to treatments and CO_2_ exposure. Changes however appeared to be due to presence of CO_2_ alone, as the secondary treatment had no effect. This is unsurprising as volume of air moved in and out of the lung should logically increase to counteract elevated CO_2_. Interestingly, within the HDE exposed animals the peak expiratory flow, peak inspiratory flow, and tidal volume measures increased with increasing ppm co-exposure to CO_2_, suggesting that when co-exposed, the increasing doses of carbon dioxide may be resulting in increases in airways responsiveness. While this would fit with the barn dust extract and CO_2_ treatment being one of the most inflammatory treatments tested here, the changes were nearly immediate within the monitoring time (within 40 min, data not shown) which may suggest very early inflammatory mechanisms could be playing a role. Overall however we would expect minimal changes to breathing due to the short time frame of the exposure.

Looking at cellular levels in the lung extracellular space we see that similar to our previous study [16], increasing CO_2_ appeared to have no effect on saline treated animals. HDE by comparison showed a trend to increases in total cells and a trend to increased monocytes with CO_2_ co-treatment, even at levels as low as 1000 ppm CO_2_, and by 5000 ppm a significant increase in total BAL cells and neutrophils in the lung. This general increased inflammatory profile is similar to our previous report [16]. A further similarity to that report was that in lung tissue histology (Fig. 5) there was no discernable changes seen with any of the treatments with or without added CO_2_. Given that this was a 6 h exposure, we did not expect to see significant histological changes. We suspect a more chronic exposure may yield different results though, given other changes in cytokines and chemokine expression.

Past work by our lab has shown that CO_2_ co-exposure did cause significant changes at the molecular level, increasing inflammatory responses to organic dusts. As LPS is a common constituent of agricultural dusts it was thought that LPS exposures and co-exposures may respond similarly to HDE. Our results showed a relatively common pattern across several cytokines with regards to LPS as compared to saline, showing significant increase in inflammatory cytokine production with LPS exposure. However, for the proinflammatory cytokines, the LPS with room air response tended to be greater than the HDE with room air response. These responses changed with co-exposure to CO_2_. For LPS, there were significant decreases in cytokines with both levels of CO_2_ co-exposures. By contrast, when HDE was co-exposed with CO_2_, at 1000 ppm there was a decreased cytokine response compared to the room air HDE + CO_2_ co-exposure, however, at 5000 ppm HDE + CO_2_ co-treatment the cytokines were greater as compared to the co-exposures to room air and 1000 ppm CO_2_. These results are similar to the BAL cellular responses, particularly with well-known monocyte and macrophage chemoattractors such as MCP-1, MIP-2, KC, TNF-a, and IL-1β [33, 34]. IL-1β should warrant particular attention given past work in ex-vivo systems with neutrophil microparticles that shows increased levels of IL-1β per microparticle with increasing CO_2_ up to 4000 ppm [17]. While we did not see this with unstimulated animals, we do see this increased IL-1B with HDE co-exposure. The similar results shown with human and mouse neutrophils in response to CO_2_ suggests the mouse model could reflect similar patterns. Their further proof of iNOS activation provides yet another avenue of exploration in future studies.

Of the cytokines tested TNF-α, MCP-1, MIP-2 and KC are all are transcriptionally controlled by NF-kB and AP-1 [35–38], suggesting possible targets for future work on mechanisms of CO_2_ inflammation. Very little is currently known about a CO_2_ “sensor” or signaling pathway in cells. Several studies have reported effects on certain proteins such as ERK and JNK activation [39]. The most promising results for immunological effects have looked at changes to the IKKa and NF-kB alternate pathway signaling (P100 and RelB) in response to CO_2_ exposure [40, 41]. These results implicate the NF-kB pathway as being important. For this reason, we decided to examine expression of receptors and regulators of this pathway associated with organic dust exposure and LPS. TLR2 and TLR4 are two of the most heavily implicated receptors associated with HDE exposure [3, 28, 29, 42]. TLR4 is also the primary receptor associated with LPS detection [43]. Interestingly we can see that TLR4 mRNA expression tended to be similar for all treatments. TLR2 on the other hand appears to exhibit a pattern of expression that follows a pattern similar to that seen with many of the proinflammatory cytokines we tested for, though other than for LPS + 1000 ppm CO_2_, the changes were not significant. As TLR2 can bind to LPS [44], the reduction in TLR2 mRNA may suggest a mechanism for reduced LPS responses with higher CO_2_ but would not explain the increased indicators of inflammation seen with HDE. An examination at the mRNA and protein level of TLR2, TLR4, as well as MD-2 and CD14 [43, 45, 46], all of which are involved in the binding of LPS to TLR2 and TLR4 [47] could assist in further delineating pathway involvements.

More recently an important upstream regulator of NF-kB signaling, TNFAIP3 (A20), was found to be vital in development of protection to asthma in rural children exposed to LPS [30]. While this may suggest that farm work may be protective against some respiratory problems, those working in concentrated animal feed operations receive high endotoxin exposure but show increased susceptibility to a variety of respiratory problems, including asthma [30]. Thus, we examined A20 mRNA expression in response to CO_2_. While there was little expression of A20 when exposed to LPS alone, there was a significant increase in expression with exposure to HDE, but this was inhibited by CO_2_ co-exposure, particularly at higher levels. While this result needs to be explored further it does suggest one way in which elevated CO_2_ may enhance HDE innate immune responses, by reducing expression of the inhibitor A20. Extrapolating to the previously mentioned study [30] such an A20 reduction may result in a non-protective exposure, if A20 is as critical to inhibiting future atopic responses as has been reported. This association of CO_2_ with worse asthma [48] and wheeze has been shown in several daycare and school studies [10, 11]. It is possible that CO2 exerts changes on innate immunity through NF-kB, but would further suggest that this may be done (or additionally modified) through further upstream pathways that may induce NF-kB (TLRs) or regulate other such inputs and NF-KB such as A20. Studies looking at blocking such regulatory pathways and their effect on NF-kB in this context would be a useful next step.

Finally, we looked at a very general heat shock protein, Hsp72 (HspA1). This particular protein has been shown to be produced in response to a broad array of stress stimuli [49]. Links between HspA1 and control of a more recently examined player in dust inflammation, HMGB1, made us interested in its induction. Interestingly, there were significant reductions in HspA1 when LPS was co-exposed with CO_2_, even at levels as low as 1000 ppm CO_2_, and a trend in HDE treated animals in the same direction. As localization of HspA1 is a strong indicator of whether it is a pro or anti-inflammatory agent, longer exposures will need to be done in the future, looking at protein expression and localization. Our initial mRNA results suggest that addition of a CO_2_ co-exposure appears to reduce HspA1 mRNA when given in conjunction with an inflammatory stimulus.

While this work shows interesting results related to co-exposures to inflammatory agents, additional work is necessary. This work extends previous findings [16] and uses well quantified and described organic dust samples. The work is limited to describing effects in female mice and similar studies need to be replicated in male animals. Further applicability to humans should also be taken with some reservation given established differences in lung anatomy between mice and what this may mean to parameters such as lung function [50]. Future studies with proteomic assessment of these inflammatory measures, assist in determining the breadth and scope of changes CO_2_ induces. Assessment of NF-kB signaling, including use of its alternate signaling pathway should be conducted. There is of course the need to assess chronic exposures, as well as situations of removing CO_2_ co-exposure at the start or end of an exposure period, to see if there is a time in which this increased CO_2_ exposure is critical to alteration of innate immunity. Several of these parameters are part of ongoing work in our lab.

While this study was to look at exposures common in animal feed facilities, there is ample evidence that people are exposed in a number of environments to elevated CO_2_ that exceed 1000 ppm [15]. This work highlights that immunological exposures may not just be a factor of the introduced inflammatory agent, but also the environmental context in which it is given. The importance of CO_2_ in respiratory effects may be particularly important where indications of respiratory symptoms in children such as wheeze, asthma [10, 11], and respiratory infection [51] could be exacerbated by increased CO_2_ exposure in their environment in addition to more commonly tested irritants. We show here that expression of mRNA of some innate immune receptors may be altered with just CO_2_ alone, but that response to inflammation can be significantly altered by these indoor CO_2_ levels at 1000 ppm or higher. Interestingly, our current study suggests that these responses will not necessarily be modified in the same manner across different inflammatory agents. This highlights the further need to deduce signaling pathways or commonalities in responses to certain immunological insults to better predict the impact of CO_2_. If response to an infection, or progression of certain chronic diseases (ex. COPD, asthma) can be impacted by elevated CO_2_, the possibility exists of ameliorating some effects of these same illnesses by improving ventilation.

## Data Availability

The datasets generated during and/or analysed during the current study are available from the corresponding author on reasonable request.
